# Integrated clinical and genomic evaluation of guadecitabine (SGI-110) in peripheral T-cell lymphoma

**DOI:** 10.1038/s41375-022-01571-8

**Published:** 2022-04-22

**Authors:** Jonathan Wong, Emily Gruber, Belinda Maher, Mark Waltham, Zahra Sabouri-Thompson, Ian Jong, Quinton Luong, Sidney Levy, Beena Kumar, Daniella Brasacchio, Wendy Jia, Joan So, Hugh Skinner, Alexander Lewis, Simon J. Hogg, Stephin Vervoort, Carmen DiCorleto, Micheleine Uhe, Jeanette Gamgee, Stephen Opat, Gareth P. Gregory, Galina Polekhina, John Reynolds, Eliza A. Hawkes, Gajan Kailainathan, Robin Gasiorowski, Lev M. Kats, Jake Shortt

**Affiliations:** 1 Monash Haematology, Monash Health, Clayton, VIC Australia; 2 Blood Cancer Therapeutics Laboratory, Department of Medicine, School of Clinical Sciences at Monash Health, Monash University, Clayton, VIC Australia; 3 Sir Peter MacCallum Department of Oncology, University of Melbourne, Parkville, VIC Australia; 4 Peter MacCallum Cancer Centre, Melbourne, VIC Australia; 5 Monash Health Imaging, Monash Health, Clayton, VIC Australia; 6 Department of Imaging, School of Clinical Sciences at Monash Health, Monash University, Clayton, VIC Australia; 7 Monash Pathology, Monash Health, Clayton, VIC Australia; 8 Department of Epidemiology and Preventive Medicine, School of Public Health and Preventive Medicine, Monash University, Melbourne, VIC Australia; 9 Biostatistics Consulting Platform, Monash University and Alfred Health, Prahran, VIC Australia; 10 Olivia Newton John Cancer Wellness and Research Centre, at Austin Health, Heidelberg, VIC Australia; 11 Transfusion Research Unit, School of Public Health and Preventive Medicine, Monash University, Melbourne, VIC Australia; 12 Haematology Department, Concord Repatriation General Hospital, Concord, NSW Australia; 13 University of Sydney, Sydney, NSW Australia

**Keywords:** Translational research, T-cell lymphoma

## Abstract

Peripheral T-cell lymphoma (PTCL) is a rare, heterogenous malignancy with dismal outcomes at relapse. Hypomethylating agents (HMA) have an emerging role in PTCL, supported by shared mutations with myelodysplasia (MDS). Response rates to azacitidine in PTCL of follicular helper cell origin are promising. Guadecitabine is a decitabine analogue with efficacy in MDS. In this phase II, single-arm trial, PTCL patients received guadecitabine on days 1–5 of 28-day cycles. Primary end points were overall response rate (ORR) and safety. Translational sub-studies included cell free plasma DNA sequencing and functional genomic screening using an epigenetically-targeted CRISPR/Cas9 library to identify response predictors. Among 20 predominantly relapsed/refractory patients, the ORR was 40% (10% complete responses). Most frequent grade 3-4 adverse events were neutropenia and thrombocytopenia. At 10 months median follow-up, median progression free survival (PFS) and overall survival (OS) were 2.9 and 10.4 months respectively. *RHOA*^*G17V*^ mutations associated with improved PFS (median 5.47 *vs*. 1.35 months; Wilcoxon *p* = 0.02, Log-Rank *p* = 0.06). 4/7 patients with *TP53* variants responded. Deletion of the histone methyltransferase *SETD2* sensitised to HMA but *TET2* deletion did not. Guadecitabine conveyed an acceptable ORR and toxicity profile; decitabine analogues may provide a backbone for future combinatorial regimens co-targeting histone methyltransferases.

## Introduction

Peripheral T-cell lymphoma (PTCL) is a rare, heterogenous malignancy with dismal outcomes at relapse. However, emerging data demonstrate the susceptibility of PTCL to epigenetically-targeted drugs [1]. The hypomethylating agent (HMA), 5’azacitidine (AZA) has been repurposed in PTCL based on mutational overlap with myelodysplasia (MDS), an HMA responsive disease [2]. Mutations of enzymes regulating DNA methylation: Ten-Eleven Translocation-2 (*TET2)*, DNA methyltransferase-3A (*DNMT3A*) and isocitrate dehydrogenase-2 (*IDH2*) are frequently perturbed in angioimmunoblastic T-cell lymphoma (AITL) and PTCL of T-follicular helper phenotype (hereafter referred to in aggregate as T-cell lymphomas of T-follicular helper origin [tTFH]) [3–7]. Interestingly, patients with *TET2*-mutated tTFH frequently exhibit clonal hematopoiesis of indeterminate potential (CHIP) and may develop clonally related myeloid neoplasms that are HMA responsive [8].

Aside from a tTFH phenotype, predictors of HMA response are poorly characterised and HMA activity in other PTCL histologies is undefined. A retrospective study of parenterally administered AZA reported an ORR of 75% in 12 patients with AITL, including a durable remission [2]. This study included predominantly older patients, many of whom had concurrent MDS and *TET2* mutations. A recent prospective trial yielded an 80% ORR in patients with tTFH when orally administered AZA (CC486) was combined with romidepsin [9]. Here the responding patients were enriched for mutations in genes regulating epigenetic processes. Although *TET2* mutations predict increased responsiveness to HMA in MDS, patients lacking *TET2, DNMT3A* and *IDH2* mutations may still respond to HMAs [10]. Moreover, we reported a rapid and durable AZA response in relapsed/refractory (RR)-AITL lacking any such mutations or copy number variations in TET-family hydroxymethylases [11].

Guadecitabine (SGI-110, Astex Pharmaceuticals Inc.) is an oligonucleotide decitabine prodrug that is resistant to metabolism by cytidine deaminase, conveying improved pharmacokinetic properties and greater in vivo DNA demethylation than decitabine [12]. Guadecitabine induced responses in patients with acute myeloid leukemia (AML) who had previously received induction therapy and in MDS patients who had failed AZA or decitabine, and was non-inferior to these agents in a phase III front-line study for frail AML patients [13, 14]. The activity of decitabine analogues is undefined in PTCL and the efficacy of single-agent AZA has not yet been prospectively reported. Here we present the results of a phase II trial of guadecitabine in PTCL. To better define response predictors, we complimented the clinical study with molecular analyses, including CRISPR/Cas9 functional genomic screening to identify epigenetic modulators of HMA response.

## Materials and methods

### Study design and patients

This was a phase II single-arm study. Sample size was pragmatically determined with 20 patients expected to be registered on the study in 24 months. The maximum standard error for the ORR after six cycles of induction was 11.2% and this was considered suitable precision for a phase II pilot study. Eligible patients were ≥18 years old with Eastern Cooperative Oncology Group (ECOG) ≤3 and histologically confirmed, treatment naïve (TN) or R/R WHO-defined PTCL. TN patients were only eligible if determined by the treating clinician as unfit for intensive chemotherapy due to comorbidities. Concurrent MDS was permitted. Exclusion criteria included: prior HMA treatment, central nervous system involvement, active second malignancy requiring therapy, uncontrolled viral infection or other medical conditions contraindicating HMA due to inadequate organ function. Corticosteroids were permitted up to 20 mg prednisolone equivalent for lymphoma-related immune manifestations up to the time of guadecitabine therapy.

The study was approved by local institutional review board (Ref: 17-0000-631A) and conducted according to the provisions of the Declaration of Helsinki and International Conference on Harmonisation Guidelines for Good Clinical Practice. All subjects provided informed consent before enrolment. This trial was registered at www.anzctr.org.au, #ACTRN12618000028202.

### Treatment

Guadecitabine was administered at 60 mg/m^2^ subcutaneously on days 1–5 every 28-days until disease progression. Cycle length could be shortened at investigator discretion for suspected disease progression between cycles provided safety thresholds were met. Treatment delays and dose reductions were permitted for grade 3–4 cytopenias unrelated to concurrent myeloid disorder or marrow involvement with lymphoma. Reasons for cessation in addition to progressive disease included unacceptable toxicity, management of comorbidity requiring treatment cessation or withdrawal of consent. G-CSF support was recommended for grade 3–4 neutropenia to maintain dose intensity. Mould-spectrum antifungal prophylaxis (posaconazole) was recommended for patients considered high-risk for invasive fungal infection based on concurrent myeloid disorder or prolonged grade 3-4 neutropenia. Antiviral (valaciclovir) and *Pneumocystis* (co-trimoxazole) prophylaxis were mandated. Rituximab was permitted for Epstein-Barr virus (EBV) reactivation according to investigator discretion.

### Outcome measures

Co-primary endpoints were investigator-assessed ORR (achievement of complete response [CR] or partial response [PR]) and safety/tolerability (defined as incidence and severity of adverse events [AEs] during the first six ‘induction’ cycles). Secondary endpoints were PFS for all patients and ORR in the subset with tTFH. PFS and OS were measured from day one of guadecitabine treatment. Responses were deemed evaluable if the patient received at least one guadecitabine dose. Duration of response (DOR) was measured from the date PR (or better) was first observed to the earlier of date of progression or death. Exploratory endpoints included assessments of potential response biomarkers. Response assessment was by Lugano criteria [15]. Adverse events were evaluated using the Common Toxicity Criteria for Adverse Events (CTCAE v4.03). Positron-emission tomography (PET)/computed tomography (CT) scans were performed after cycles two, four and six and as clinically indicated thereafter until progression or withdrawal from study. PET/CT scans were centrally reviewed, and total metabolic tumor volume (TMTV) assessed using the 41% SUVmax method [16]. Bone marrow examinations were performed at baseline for staging and to diagnose/exclude concurrent myeloid disorder. Repeat bone marrow biopsies were protocolised for response assessments. Patients withdrawing from the study for reasons other than disease progression had time-to-event endpoints censored at the time of study withdrawal.

### Statistical analyses

OS and PFS were evaluated by Kaplan-Meier analysis and exploratory comparisons used both the Wilcoxon and Log-Rank tests. A landmark analysis of OS in responders versus non-responders was restricted to patients who were alive at two months [17]. Median potential follow-up was estimated by reversing the censor indicator in a Kaplan-Meier analysis of OS [18]. Categorical variables were compared using Fisher’s exact test. 50% lethal concentrations (LC_50_) were calculated using non-linear regression. Analyses were performed using GraphPad Prism (Version 9.2.0) and SAS Software (Version 9.4).

### Tumor and plasma DNA mutational analyses

DNA from buccal swabs and Streck Cell-Free BCT^®^ tubes (La Vista, NE, USA) was purified using commercial isolation kits (Qiagen, Venlo, Netherlands) and quality metrics performed using Bioanalyzer (Agilent Technologies, CA, USA). NGS libraries were constructed using Agilent XTHS reagents and protocols incorporating unique molecular barcoding (Agilent Technologies, CA, USA). Primary tumors were sequenced using Agilent SureSelect Human All Exon V7. For cell free tumor DNA (ctDNA) and buccal analysis, a bespoke bait capture design was used encompassing 36 genes and a CNV backbone. Total bait coverage for this smaller capture set was 435 kb and included genes as listed in Supplementary Table S1. Sequencing was performed on a NovaSeq 6000 (Illumina, SP flow cell, 2 x 150bp chemistry) yielding a mean sequencing depth (across baited gene regions prior to UMI deduplication) of 35,400 and 178 for ctDNA and buccal libraries respectively. Data processing of FASTQ files was performed using an in house bioinformatic pipeline. Variant calls (identified by *VarDict* [19]) were manually curated by inspecting BAM files in Integrative Genomics Viewer [20]. For ctDNA sequencing at the achieved sequencing depths, sensitivity of SNV detection was confirmed at a variant allele frequency (VAF) of ~0.2%, determined by several criteria including spike-in simulation using B*AMSurgeon* [21]. Copy number analysis was performed using CNVkit [22] and mutational plots were visualized using GenVisR [23].

### Data sharing statement

De-identified patient data will be shared upon request within three years of publication subject to a data sharing agreement and an ethically approved research proposal. The accession number for the RNA sequencing data reported in this paper is GEO: GSE188571.

For detailed description of methods, including expression profiling and CRISPR screening, please refer to methodology supplement.

## Results

### Patients and treatment

Between June 2018 and January 2020, 20 patients were enrolled. Baseline patient characteristics are summarised in Table 1. Eighty percent had tTFH and 90% had R/R disease. Two patients were TN, one of whom had concurrent chronic myelomonocytic leukemia (CMML). Subjects received a median of 3.5 cycles (range 1–16) with a median dose of 60 mg/m^2^ and cycle length of 28d per induction cycles (Supplementary Fig. S1). Thirteen patients received G-CSF support and six patients received antifungal prophylaxis. Six patients had EBV viraemia at baseline (median plasma EBV viral load 1628 copies/mL; range 400–257538 copies/mL); the patient with highest viral load received two doses of rituximab from cycle one of guadecitabine with resolution of viraemia. One patient who was EBV PCR negative at baseline developed EBV reactivation after cycle eight and received one dose of rituximab with improvement in viraemia (EBV viral load reduction from 262079 to 41038 copies/mL) before withdrawal from the protocol with progressive lymphoma.

**Table 1:** Pre-treatment patient characteristics.

Characteristic	Patients (*n* = 20)
**Age, median [range]**	65 [51–81]
**Gender,** ***n*** **(%)**	
Male	14 (70)
Female	6 (30)
**ECOG performance status,** ***n*** **(%)**	
0	7 (35)
1	7 (35)
2	3 (15)
3	3 (15)
**Extent of disease at study entry,** ***n*** **(%)**	
Stage III	7 (35)
Stage IV	13 (65)
Extra nodal disease	13 (65)
Bone marrow involvement	3 (15)
Cutaneous involvement	5 (25)
Other (GIT, pleural, peritoneal, lung, liver)	5 (25)
Elevated LDH	9 (45)
**International Prognostic Index,** ***n*** **(%)**	
0–1	0 (0)
2	8 (40)
3–5	12 (60)
**PTCL subtype,** ***n*** **(%)**	
Angioimmunoblastic T-cell lymphoma	11 (55)
PTCL-TFH	5 (25)
PTCL-NOS	2 (10)
Anaplastic large cell lymphoma (ALK -ve)	1 (5)
MEITL	1 (5)
**Concurrent myeloid disorder,** ***n*** **(%)**	
Chronic myelomonocytic leukemia	1 (5)
**Prior lines of therapy,** ***n*** **(%)**	
0 (Treatment naïve)	2 (10)
1	2 (10)
2	6 (30)
3	1 (5)
> 3	9 (55)
median prior lines [range]	3.5 [1–9]
Prior autologous stem cell transplant	8 (40)
**Type of prior systemic therapy,** ***n*** **(%)**	
Any chemotherapy	18 (90)
CHO(E)P-like	16 (80)
Brentuximab vedotin	2 (10)
Romidepsin	6 (30)
Pralatrexate	6 (30)
Methotrexate	2 (10)
Cyclosporin	2 (10)
**Prior radiotherapy,** ***n*** **(%)**	1 (5)

*ECOG* Eastern Cooperative Group, *GIT* Gastrointestinal tract, *LDH* Lactate dehydrogenase, *PTCL* Peripheral T-cell lymphoma, *TFH* T-follicular helper, *NOS* Not otherwise specified, *ALK* Anaplastic lymphoma kinase, *MEITL* Monomorphic epitheliotropic intestinal T-cell lymphoma, *CHO(E)P* Cyclophosphamide, doxorubicin, vincristine, etoposide, prednisolone.

### Safety

Adverse events are summarised in Table 2. Adverse events of grade 3–4 occurred in 90% of patients, most commonly neutropenia (50%) and thrombocytopenia (30%). There were no AEs related to clinically significant bleeding events. Neutropenia was prominent in early cycles, with 20% of the cohort developing febrile neutropenia in cycle one. The incidence of neutropenia in subsequent cycles was mitigated by protocol-defined dose delays (30% of patients with at least one cycle delayed ≥ 1 week) and/or dose reduction (40% of patients) during the first six cycles (Supplementary Fig. S1). Twenty-six severe AEs occurred in 13 patients (most commonly management of non-neutropenic infections, febrile neutropenia and fevers without documented infection [31%, 27% and 15% of severe AEs respectively]). Significant non-haematological toxicities were infrequent, and similar in nature to those expected for low intensity HMA therapy. There were no grade 5 AEs.

**Table 2:** Grading (severity) of adverse events (AEs) regardless of relationship to study treatment by preferred term during the first six ‘induction’ cycles (*n* = 20).

Rank	Adverse event	Any grade		Grade 3		Grade 4	
		*n*	%	*n*	%	*n*	%
	Any preferred term	20	100	7	35	11	55
1	Neutropenia	12	60	4	20	6	30
2	Constipation	7	35	0	0	0	0
3	Thrombocytopenia	7	35	3	15	3	15
4	Fatigue	6	30	0	0	0	0
5	Febrile neutropenia	6	30	3	15	3	15
6	Fever	6	30	3	15	0	0
7	Oedema	5	25	0	0	0	0
8	Rash	5	25	0	0	0	0
9	Anaemia	4	20	3	15	1	5
10	Diarrhoea	4	20	0	0	0	0
11	URTI	4	20	0	0	0	0
12	Abdominal pain	3	15	0	0	0	0
13	Nausea	3	15	0	0	0	0
14	Bone pain post G-CSF	2	10	0	0	0	0
15	Bruising	2	10	0	0	0	0
16	Cough	2	10	1	5	0	0
17	Headache	2	10	0	0	0	0
18	Pruritis	2	10	0	0	0	0
19	Rhinovirus infection	2	10	0	0	0	0
20	Sore throat	2	10	0	0	0	0
21	UTI	2	10	0	0	0	0

There were no grade 5 AEs. *URTI* upper respiratory tract infection, *G-CSF* granulocyte colony stimulating factor, *UTI* urinary tract infection.

### Efficacy

**Fig. 1:**
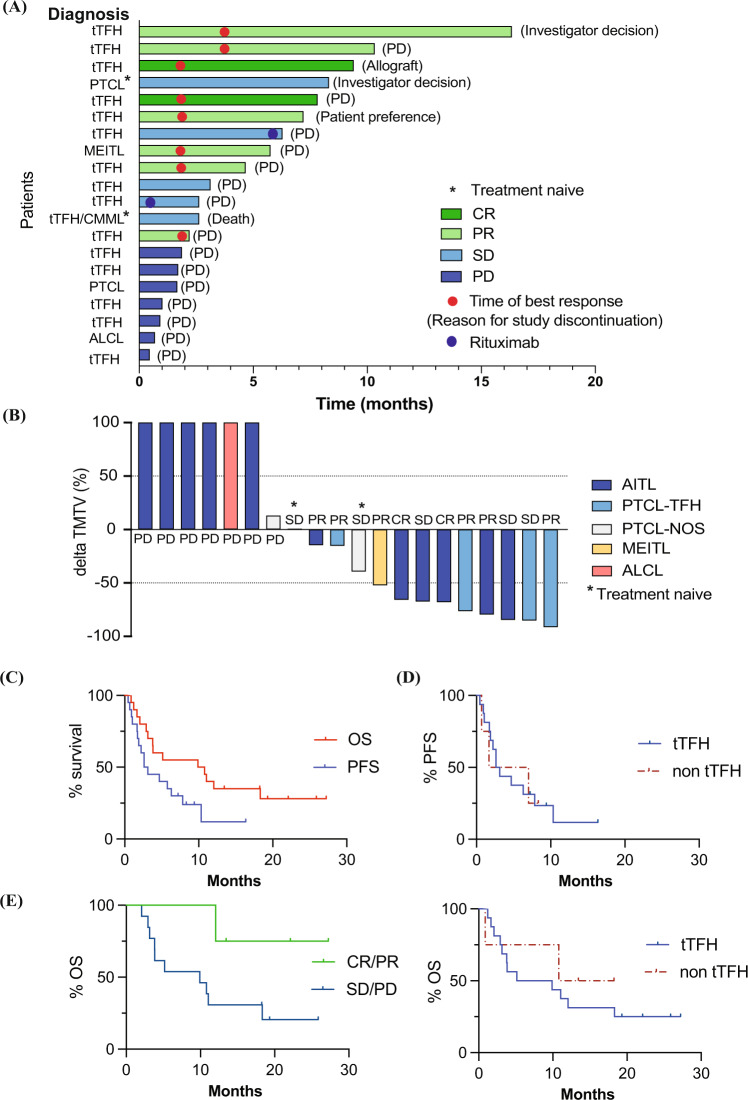
Treatment response summary. **A** Swimmer plots demonstrating progress of subjects treated for T-cell lymphoma with guadecitabine from treatment initiation to discontinuation. **B** Change in total metabolic tumor volume at time of maximal clinical response relative to baseline. Those patients who withdrew from the study prior to cycle 2 FDG-PET assessment are arbitrarily designated + 100%. **C** PFS and OS of guadecitabine-treated patients (**D**) PFS (top panel) and OS (lower panel) of guadecitabine-treated patients stratified by tTFH status. **E** Landmark analysis of OS from 2 months for response (PR + CR) *vs* non-response (SD + PD). PTCL Peripheral T-cell lymphoma, tTFH T-cell lymphoma of T-follicular helper origin, MEITL Monomorphic epitheliotropic intestinal T-cell lymphoma, CMML Chronic myelomonocytic leukemia, ALCL Anaplastic large cell lymphoma, CR Complete response, PR Partial response, SD Stable disease, PD Progressive disease.

Responses were seen in eight (40%) of 20 evaluable patients with two (10%) patients achieving CR (Table 3). Both CRs and 5/6 PRs occurred in subjects with tTFH (Fig. 1A, B). There was one death on protocol treatment due to progressive disease after three cycles with palliative measures in place. This subject was a TN 78-year-old who commenced the study with concurrent AITL and CMML and ECOG = 3. In addition to the 40% of subjects with an objective response, three subjects demonstrated stable disease (SD) with a >50% reduction in TMTV (Fig. 1B) and two with a best response of SD remained on study beyond six cycles. 15/20 patients discontinued the trial due to progressive disease, four prior to first response assessment. Two patients discontinued the trial protocol to manage pre-existing medical comorbidities (coronary disease and cutaneous squamous cell carcinomas respectively). One subject withdrew from the protocol to undergo allogeneic transplant after achieving a CR to guadecitabine treatment.

**Table 3:** Treatment response summary.

Best response	All patients (*n* = 20) *n* (%)	tTFH (*n* = 16) *n* (%)
**Complete response**	2 (10%)	2 (13%)
**Partial response**	6 (30%)	5 (31%)
**Overall response rate** (CR + PR)	8 (40%)	7 (44%)
**Stable disease**	5 (25%)	4 (25%)
**Progressive disease**	7 (35%)	5 (31%)
**Disease control rate** (CR + PR + SD > 6 cycles)	10 (50%)	8 (50%)

*CR* Complete response, *PR* Partial response, *SD* Stable disease, *tTFH* T-cell lymphoma of T-follicular helper phenotype.

At the time of data analysis, with an estimated median potential follow-up of 22 months (range 13-27) the median PFS and OS were 2.9 (95% CI: 1.6–7.9) and 10.4 (95% CI: 2.9–18.3) months respectively (Fig. 1C). There was no significant difference in ORR, median PFS and OS between tTFH and other histologies (Fig. 1D, Table 3). The estimated median time to best response was 11.7 (95% CI: 1.9–not reached) months and 2.0 (interquartile range: 1.8–3.1) months in responders. Median duration of response (DOR) was 6.0 (95% CI: 0.7–not reached) months. Patients attaining an objective response appeared to demonstrate a significant OS advantage over non-responders (Supplementary Fig. S2, *n* = 20, median OS not reached versus 3.5 months; *p* < 0.001) and this was also evident in the landmark analysis from 2 months (Fig. 1E, *n* = 17, median OS not reached versus 9.9 months; *p* = 0.073).

### Translational and exploratory endpoints

**Fig. 2:**
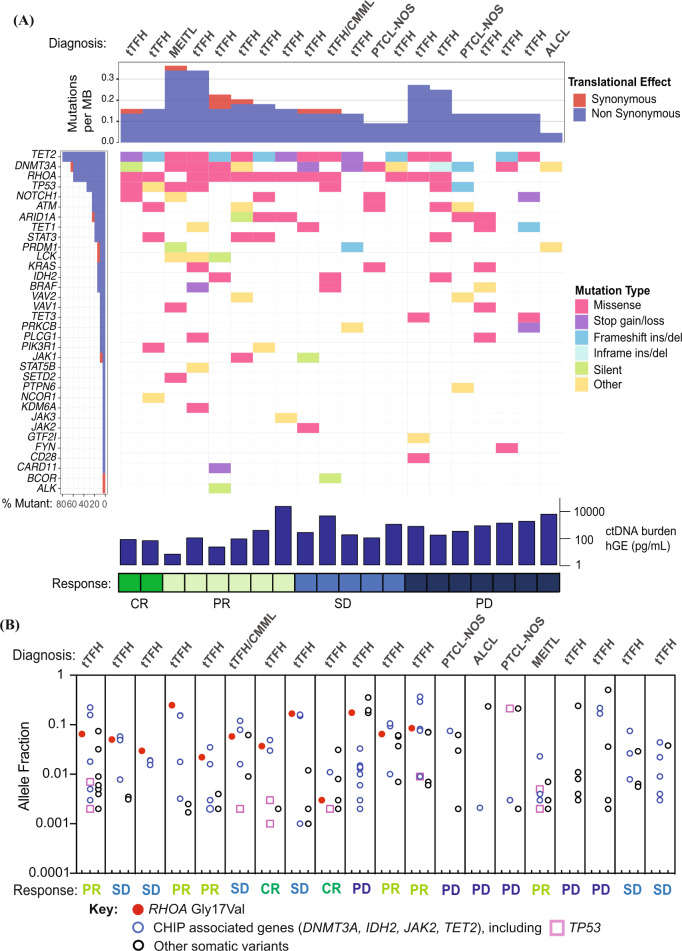
Mutation analysis of ctDNA plasma. **A** Co-mutation plot for variants detected in ctDNA at study entry clustered according to best clinical response. **B** Variant allele fractions for *RHOA, TP53* and CHIP associated mutations from individual subjects at trial baseline. *RHOA* mutated cases are represented to the left of the figure. ctDNA cell free tumor DNA, PTCL Peripheral T-cell lymphoma, tTFH T-cell lymphoma of T-follicular helper origin, MEITL Monomorphic epitheliotropic intestinal T-cell lymphoma, CMML Chronic myelomonocytic leukemia, ALCL Anaplastic large cell lymphoma, CR Complete response, PR Partial response, SD Stable disease PD Progressive disease, CHIP Clonal hematopoiesis of indeterminate potential, MB Megabase, hGE Human genome equivalents.

To correlate histological diagnoses and clinical responses with the mutational profile of lymphomas, ctDNA was analysed on plasma collected immediately prior to guadecitabine treatment. Consistent with prior reports in tTFH, most patients harbored mutations in genes regulating DNA methylation and other genes associated with concurrent clonal hematopoiesis, and a range of copy number variations (Fig. 2 and Supplementary Figs. S3, 4) [8]. The prevalence of *TET2*, *DNMT3A* and *IDH2* mutations was 80%, 60% and 15% respectively, including 50% compound mutants for *TET2* plus *DNMT3A* and/or *IDH2* concurrently. Multiple mutated *TET2* alleles (median = 2; range 1–15 unique variants) were detected in the same patients, with variant allele frequencies (VAF) spread between an approximation of putative truncal lymphoma lesions (e.g., *RHOA*^*G17V*^) and the limit of detection of the assay (~0.2%; Fig. 2B). All responses occurred in *TET2* mutated cases, whereas none of the four *TET2* wild type cases responded (*p* = 0.12). However, there was no difference in ORR or PFS in patients with *TET2*, *DNMT3A* or *IDH2* mutations compared to those who were wild type at these loci (Supplementary Table S2). *RHOA*^*G17V*^ mutations, which were present in 60% of the cohort, were associated with improved PFS (median 5.47 *vs*. 1.35 months; Wilcoxon *p* = 0.02, Log-Rank *p* = 0.06; Supplementary Table S2 and Fig. S5). Responses were not precluded by *TP53* mutations (ORR 57% for *TP53* mutant *vs*. 31% for *TP53* wild-type disease; *p* = 0.36; Supplementary Tables S2, 3), with both CRs occurring in patients with *TP53* variants.

### Guadecitabine upregulates gene expression signatures associated with inflammation and apoptosis

**Fig. 3:**
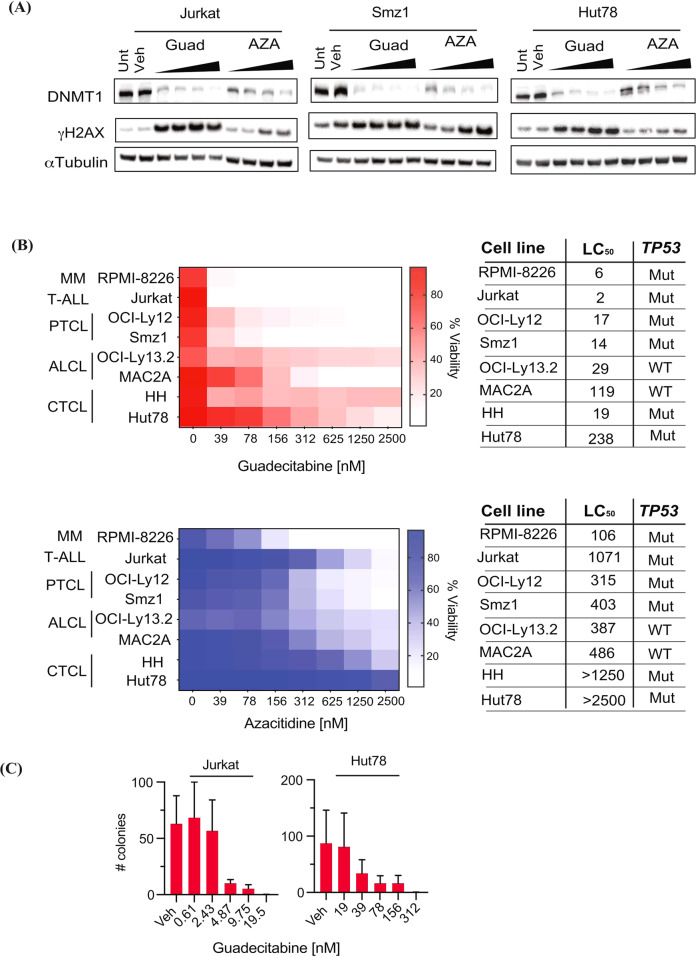
In vitro activity of guadecitabine and azacitidine versus T-cell lymphoma lines. **A** Western blot of DNMT1 expression and γH2AX phosphorylation for cells treated for 72 h with 39-312 nM of HMA. αTubulin is provided as a loading control. Results are representative of 3 independent experiments. **B** Heat map of cell viability (propidium iodide exclusion) following 5 days treatment with guadecitabine or azacitidine with viability analysis performed on day 7. The subtype of lymphoma is annotated to the left of each heat map and LC_50_ and *TP53* mutation status for each cell line are tabulated to the right. The RPMI-8226 myeloma cell line is provided as an HMA sensitive positive control. Results are the median of 3 independent experiments. **C** Colony forming assay for cells treated with guadecitabine or vehicle control for 72 h prior to plating in soft agar. Bars represent median colony counts (+/− SEM; *n* = 3 independent experiments) following 21 days culture. Unt Untreated, Veh Vehicle control, Guad Guadecitabine, AZA Azacitidine, MM Multiple myeloma, T-ALL T-cell acute lymphoblastic leukemia, PTCL Peripheral T-cell lymphoma, ALCL Anaplastic large cell lymphoma, CTCL Cutaneous T-cell lymphoma, LC_50_, 50% lethal concentration, Mut Mutated, WT Wild type.

**Fig. 4:**
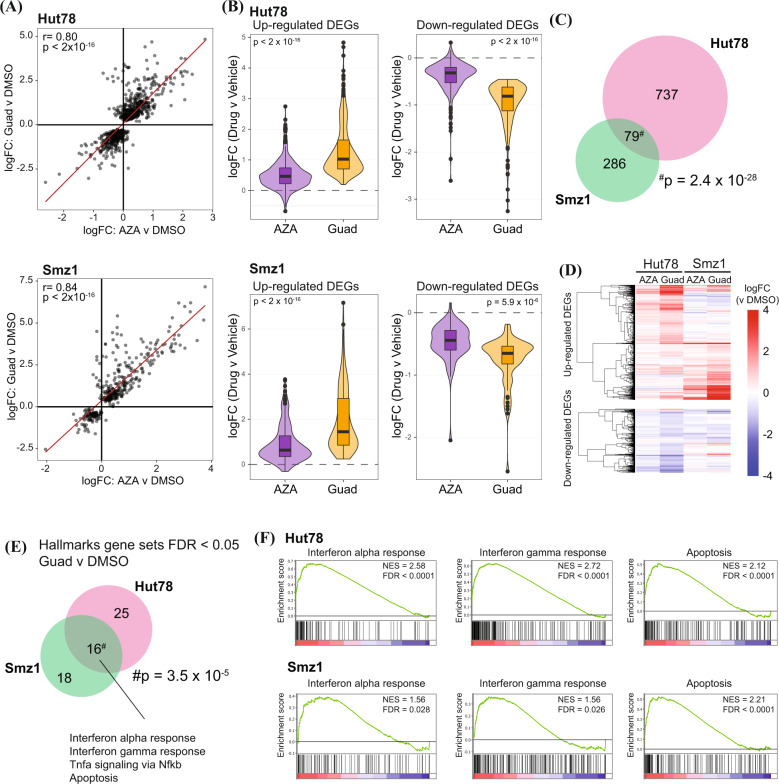
Expression profiling of HMA-treated T-cell lymphoma cells. **A** Scatterplot correlating significant DEGs (FDR < 0.05) (RNA Seq) induced by guadecitabine (100 nM) or AZA (100 nM) in Hut78 (upper panel) or Smz1 (lower panel) for 72 h of drug treatment, relative to vehicle (DMSO). **B** Violin plots of significant DEGs (FDR < 0.05 and |logFC | > 0.5) induced by either AZA or Guadecitabine in Hut78 (upper panel) or Smz1 (lower panel). **C** Venn diagram showing overlap between differentially expressed genes induced by either Guadecitabine or AZA (FDR < 0.05) in Hut78 and Smz1 cells. *P* value from hypergeometric analysis is shown. **D** Heatmap of significantly DEGs (FDR < 0.05 and |logFC | > 0.5) induced by guadecitabine or AZA, relative to vehicle, in either Hut78 or Smz1 cells. **E** Venn diagram showing overlap in Hallmark gene sets that were significantly enriched in Hut78 and SMZ1 cells treated with guadecitabine. **F** Enrichment plots of selected gene sets from (**E**). AZA Azacitidine, Guad Guadecitabine, DMSO Dimethylsulfoxide DEG Differentially expressed genes, FDR False discovery rate.

The size of the clinical cohort limited statistical power to investigate response predictors. To further elucidate response predictors, including those applicable to non-tTFH disease, we investigated HMA activity in T-cell lymphoma lines of various histological subtypes (including anaplastic large cell lymphoma, PTCL-NOS and cutaneous T-cell lymphoma). These cell lines are wild type at *RHOA* and *TET2* loci, and the majority are *TP53* disrupted (Fig. 3) [24]. Five-day guadecitabine dosing demonstrated broad and potent cytotoxicity with low nM LC_50s_ approximately 10-50-fold more potent than AZA at concentrations correlating with DNMT1 depletion (Fig. 3A, B). Guadecitabine potently reduced T-cell lymphoma clonogenicity (Fig. 3C). 3′RNAseq was performed on AZA and guadecitabine-treated Hut78 and Smz1 cells using low equimolar concentrations of both drugs after 72 h treatment (at which time there was minimal impact on cell viability; Supplementary Fig. S6). We identified differentially expressed genes (DEGs) and compared drug induced transcriptional changes across all conditions. There was high correlation between guadecitabine and AZA DEGs, indicating that both drugs exert similar cellular effects (Fig. 4A and Supplementary Tables S4, 5). Consistent with cytotoxicity data, guadecitabine was more potent than AZA at perturbing gene expression, with both up- and down-regulated genes demonstrating a greater magnitude of change in guadecitabine-treated cells (Fig. 4B). Guadecitabine or AZA induced and repressed similar genes in both Hut78 and Smz1 cells (hypergeometric *P* value 2.4 × 10^−28^), although the former were more sensitive to treatment as evident from the number of DEGs that reached statistical thresholds (Fig. 4C, D).

Gene set enrichment analysis was performed to identify biological pathways that were altered by HMA treatment in malignant T cells [25]. Concordant with previous reports in solid tumours [26, 27] and AML [28], guadecitabine upregulated pro-inflammatory signaling, including transcriptional signatures associated with type I and II interferons, TNF-α and the JAK/STAT pathways (Fig. 4E, F and Supplementary Table S6). Many genes with increased expression, such as *B2M*, *TAP1* and *CXCL11*, have been positively correlated with improved responses to immune checkpoint blockade (Supplementary Tables S4, 5) [29, 30]. Although both Hut78 and Smz1 cells carry nonsense and missense mutations in *TP53* respectively [24], apoptosis-related transcriptional programs were nonetheless induced by guadecitabine (Fig. 4E, F). Together, these data highlight that guadecitabine induces broad transcriptional changes in malignant T cells, especially among pro-inflammatory genes and apoptotic pathways.

### CRISPR/Cas9 screening identifies SETD2 as a guadecitabine sensitiser gene

**Fig. 5:**
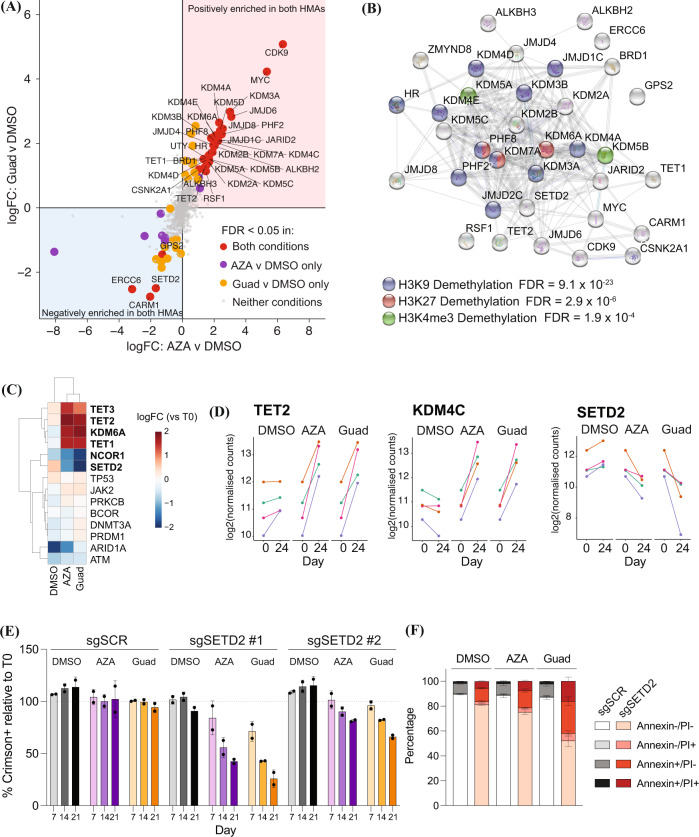
CRISPR/Cas9 screen targeting epigenetic regulators that modulate response HMAs. **A** CRISPR/Cas9 knockout screen in Hut78 cells using a custom library targeting ~900 epigenetic regulators. Scatterplot showing correlation of enrichment of genes in the presence of guadecitabine (150 nM) or AZA (300 nM) relative to DMSO after 24 days of culture. Gene enrichment was determined by MAGeCK. **B** STRING network analysis showing known interactions between proteins encoded by genes that increased or decreased sensitivity of Hut78 cells to HMA treatment. **C** Heatmap showing change in representation (logFC) of the subset of genes represented in both the CRISPR/Cas9 screen and the ctDNA panel performed on clinical trial patients (*p* < 0.05 in bold type). **D** Log2 normalised read counts for individual sgRNAs targeting the indicated genes between day 0 and 24 of the screen. **E** Competitive proliferation assay using Hut78 cells transduced vectors expressing the indicated sgRNAs and co-expressing the Crimson reporter. Cells were treated with DMSO, guadecitabine (100 nM) or AZA (800 nM). Error bars indicate mean ± s.d. from 2 biological replicates. **F** Cells were treated with DMSO, guadecitabine (100 nM) or AZA (800 nM) for 72 h and apoptosis was quantified by FACS using Annexin/PI staining. Error bars indicate mean ± s.d. from 3 biological replicates. sg, short guide RNA, SCR Scrambled AZA Azacitidine, Guad Guadecitabine, DMSO Dimethylsulfoxide, FDR False discovery rate, PI Propidium iodide.

To determine genes in which loss of function mutations could modulate sensitivity to HMAs, we performed CRISPR/Cas9 knockout sensitisation and resistance screening in Hut78 cells. Hut78s were selected as they demonstrated reduced *de novo* sensitivity to HMA relative to other cell lines (Fig. 3), thus enhancing the potential to detect sensitizer genes. As PTCL are enriched for mutations in genes regulating epigenetic processes, we designed a custom sub-library of short guide (sg) RNAs targeting ~900 genes with epigenetic functions (Supplementary Table S7). Each gene was targeted by 4 independent sgRNAs and the library also contained ~300 non-targeting control guides. The library was transduced into Hut78/Cas9 cells and cultured in guadecitabine (150 nM), AZA (300 nM) or DMSO for 24 days, maintaining a minimum representation of 2000x throughout the experiment. We used next generation sequencing to quantify sgRNA frequency after initial selection of transduced cells (T0) and then in each of the cultures after treatment (T24) and applied the MAGeCK algorithm to identify guides and genes that were negatively or positively selected over time or in the presence of HMAs [31]. As expected, most non-targeting sgRNAs were maintained during culture in all conditions whereas guides targeting pan-essential genes as defined by the cancer dependency map [32] were depleted in the DMSO condition (Supplementary Fig. S7A). Comparison of enrichment and depletion of genes in the presence and absence of the two HMA chemotypes revealed a striking correlation between guadecitabine and AZA (Fig. 5A and Supplementary Table S8). Taken together, these findings validate the performance of the screen and support a common mechanism of action of both drugs.

Inspection of the top HMA resistance and sensitisation hits revealed enrichment of enzymes regulating histone H3 methylation (Fig. 5A–C). Notably, we identified genes that encode proteins with opposing enzymatic activity that had opposite effects on HMA sensitivity as exemplified by SETD2 and KDM4C that catalyse H3K36 methylation and demethylation respectively [33]. The finding that inactivation of SETD2 sensitizes lymphoma cells to guadecitabine is consistent with our clinical observation, with the only patient on study carrying a *SETD2* mutation responding to treatment. Interestingly, silencing of *TET2* and *DNMT3A*, genes that are associated with increased sensitivity to HMAs in MDS and AML [10, 28, 34], did not produce the expected phenotype. All four *TET2*-targeting guides in the library were enriched in both guadecitabine and AZA treatment (Fig. 5D), whereas *DNMT3A* guides showed no consistent pattern (not shown).

We focussed on the SETD2 phenotype as inactivating *SETD2* mutations are recurrent in clinically important subtypes of PTCL that may be underrepresented in clinical trials due to their rarity (e.g., Monomorphic epitheliotropic intestinal T-cell lymphoma [MEITL], γδ hepatosplenic T-cell lymphoma [γδ HSTCL] and Sézary syndrome) [7, 35]. Competitive proliferation assays with independent *SETD2* guides confirmed that reduced *SETD2* expression sensitized to HMAs (Fig. 5E). We next generated clonal cell lines from bulk *SETD2* and non-targeting sgRNA transduced cells (sgSCR). SETD2 is a non-redundant methyltransferase that is the only known enzyme capable of trimethylating H3K36 in vivo [36]. In both *SETD2* mutant clones, SETD2 loss correlated with reduced H3K36me3 (Supplementary Fig. S7B). We used clones sgSCR_C1 and sgSETD2_C1 and analysed the effects of HMA treatment on proliferation and cell death (Fig. 5F and Supplementary Fig. S7C). sgSETD2_C1 cells proliferated at comparable rates to sgSCR_C1 cells in vehicle control. In contrast, while 72 h of guadecitabine or AZA treatment had minimal impact on the proliferation or survival of sgSCR_C1 cells, both drugs reduced proliferation and caused apoptosis of sgSETD2_C1 cells. Thus, SETD2 deletion does not affect the viability of Hut78 cells yet renders them sensitive to guadecitabine.

## Discussion

Our study provides the first prospective data of a single agent decitabine analogue in a predominantly R/R PTCL population with a 40% ORR and acceptable toxicity, most notably early neutropenia. The rate of neutropenia was higher than may have been anticipated for a cohort of patients where the majority (19/20) did not have a concurrent myeloid disorder. We hypothesise that this reflects the increased potency of guadecitabine relative to other hypomethylators. PTCL is a heterogenous disease group and tTFH disease appears more sensitive to epigenetic therapies [1]. Retrospective studies have reported encouraging responses to AZA in AITL, including patients with clonally related myeloid neoplasms [2]. Recent prospective trials indicate a high response rate in tTFH patients treated with CC486 combined with romidepsin [9]. Our data supports these studies with an improved PFS in the subgroup of guadecitabine-treated patients with *RHOA*^*G17V*^ mutations where *RHOA*^*G17V*^ is a hallmark of tTFH disease [3, 6, 7] and histological diagnosis may misallocate disease biology [37]. The results of a randomised phase III study comparing CC486 to investigator choice in RR-AITL are keenly awaited (clinicaltrials.gov/ct2/show/NCT03703375).

Mutations of ‘epigenetic regulators’, when considered in aggregate, associated with clinical responses in CC486/romidepsin treated patients [9] but specific indicators of response to HMA are lacking. Such mutations are enriched in tTFH but are also represented across the spectrum of both PTCL and CTCL [7]. The rarity of certain sub-entities may preclude robust representation in prospective studies. We previously reported a rapid and durable (>5 years) remission following AZA in a RR-AITL patient with *TP53* disruption and no mutation or copy number variation in TET family hydroxymethylases, *DNMT3A* or *IDH2* [11]. Similarly, mutations of these enzymes did not appear to predict responses to guadecitabine in the present study, although the low number of ‘triple mutation negative’ cases in our cohort limits this assessment. The use of ctDNA rather than tumour samples for mutational profiling requires interpretation with caution as this analysis is highly sensitive for the presence of concurrent CHIP. However, we posit where CHIP mutations are present in ctDNA at a VAF that approximates non-CHIP and lymphoma-specific mutations (e.g., *RHOA*^*G17V*^) it is likely that they are represented within the lymphoma cells; this assertion is supported by high concordance with genomic studies performed on the subset of patients on tumour tissue. Interestingly, *TET* deletion in the correlative CRISPR/Cas9 screen did not sensitize to HMA. We conclude that the absence of variants in these genes should not preclude HMA treatment, particularly in patients with tTFH.

Perturbation of *TP53* is an important determinant of chemotherapy resistance and is enriched in PTCL patients failing frontline therapy [38]. Guadecitabine demonstrated potent in vitro activity versus T-cell lymphoma cell lines irrespective of *TP53* status and *TP53* mutations did not preclude responses in this trial. This is consistent with experience in myeloid disease, where HMA responses are *TP53* independent [10]. Interestingly, guadecitabine evoked the transcriptional signature of *TP53* activation and increased γH2AX in *TP53* mutant PTCL in vitro. This suggests HMA-based interventions should be prioritised in PTCL patients with *TP53* mutations.

The only response in non-tTFH in our study was a subject with R/R MEITL who achieved a PR with >50% TMTV reduction and 6 months of disease control. *SETD2* is recurrently mutated in MEITL and more frequently so than in type 1 enteropathic T-cell lymphoma [35]. We prioritised *SETD2* for validation as one of the top sensitizers from our CRISPR screen. Interestingly, the *SETD2* mutant patient in the CC486/romidepsin study responded to therapy and the only type 1 EATL patient, who had *KDM6A*-mutant disease, did not [9]. This data provides rationale for further characterisation of the predictive role of *SETD2* mutation and H3K36me3 status in other PTCL subtypes. More broadly, unbiased genetic studies could help identify subsets of PTCL beyond tTFH for prioritisation in HMA trials.

The 40% ORR and median 2.9-month PFS in our study are comparable to that of other single-novel agent RR-PTCL studies [39, 40] but lower than the 75% ORR in retrospective series of AZA-treated AITL reported by Lemonnier et al. [2]. This may be explained by differential patient selection, particularly the exclusively AITL histology and high proportion of subjects with concurrent myeloid neoplasm in that series. AZA may also possess distinct mechanistic activity relative to guadecitabine in T-cell disease. For example, in contrast to AZA, decitabine analogues have no effect on RNA cytosine methylation and this is known to contribute to AZA responses and resistance in AML [41].

In conclusion, the ORR and toxicity profile of guadecitabine in our relatively heavily pre-treated and comorbid patient cohort were encouraging. However, the PFS and DOR were suboptimal and indicate that future studies with decitabine analogues should pursue a combinatorial approach. Our transcriptional profiling and CRISPR screen data suggest immune checkpoint inhibitors and histone methyltransferase inhibitors would rationally combine with decitabine analogues. Although guadecitabine is no longer being developed for myeloid disease, the recent registration of an orally available decitabine formulation [42] provides an alternative and convenient backbone for future studies in biologically rational PTCL subgroups including tTFH and *SETD2* mutated lymphoma.

## Supplementary information

Supplementary Figures

Methodology Supplement

Supplementary Table S1

Supplementary Table S2

Supplementary Table S3

Supplementary Table S4

Supplementary Table S5

Supplementary Table S6

Supplementary Table S7

Supplementary Table S8
