# Supplementary material for: Integrated clinical and genomic evaluation of guadecitabine (SGI-110) in peripheral T-cell lymphoma
Source: Leukemia. 2022 Apr 22;36(6):1654–65. doi: 10.1038/s41375-022-01571-8 (PMC9162925; doi:10.1038/s41375-022-01571-8)
Supplement: Supplementary file 1 — Supplementary Figures [file 41375_2022_1571_MOESM1_ESM.pdf]

(A)

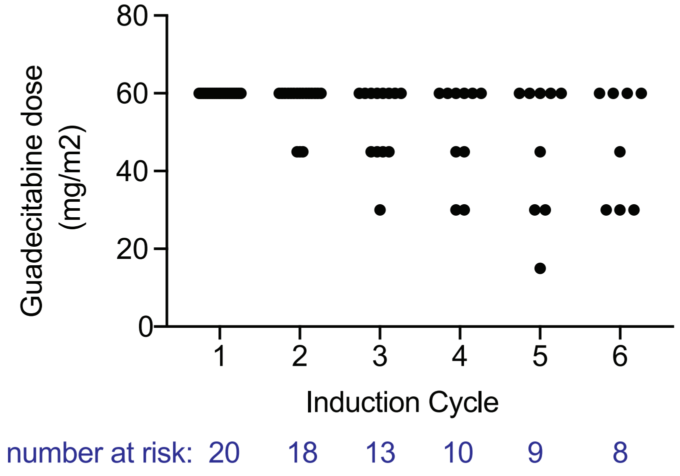

(B)

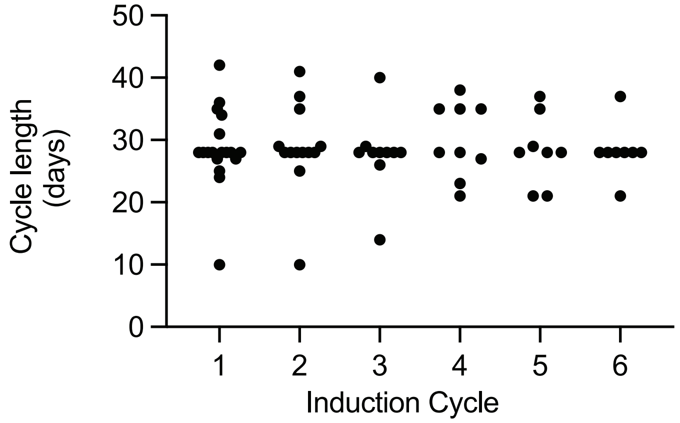

Supplementary Figure S1. (A) Guadecitabine dose level and (B) cycle length for individual subjects treated during the first six (induction) cycles.

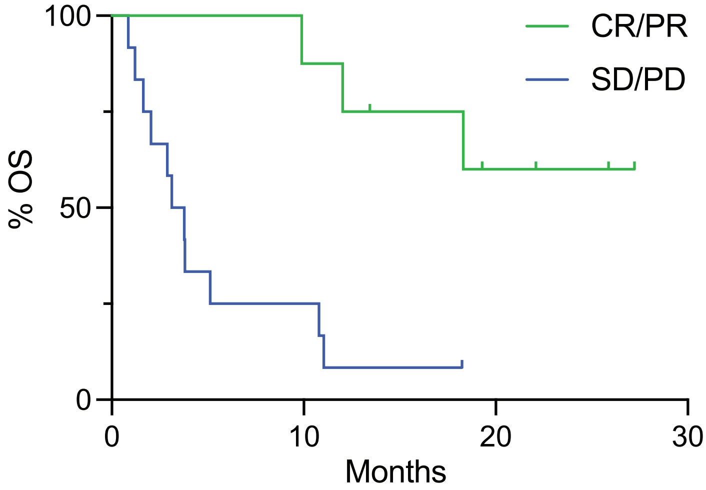

Supplementary Figure S2. Overall survival (OS) for guadecitabine-treated patients stratifed by clinical response (complete response [CR] and partial response [PR]) versus non-response (stable disease [SD] and progressive disease [PD]). Median OS not reached versus 3.5 months; $p < 0.001$ Wilcoxon, $p < 0.001$ Log-Rank.

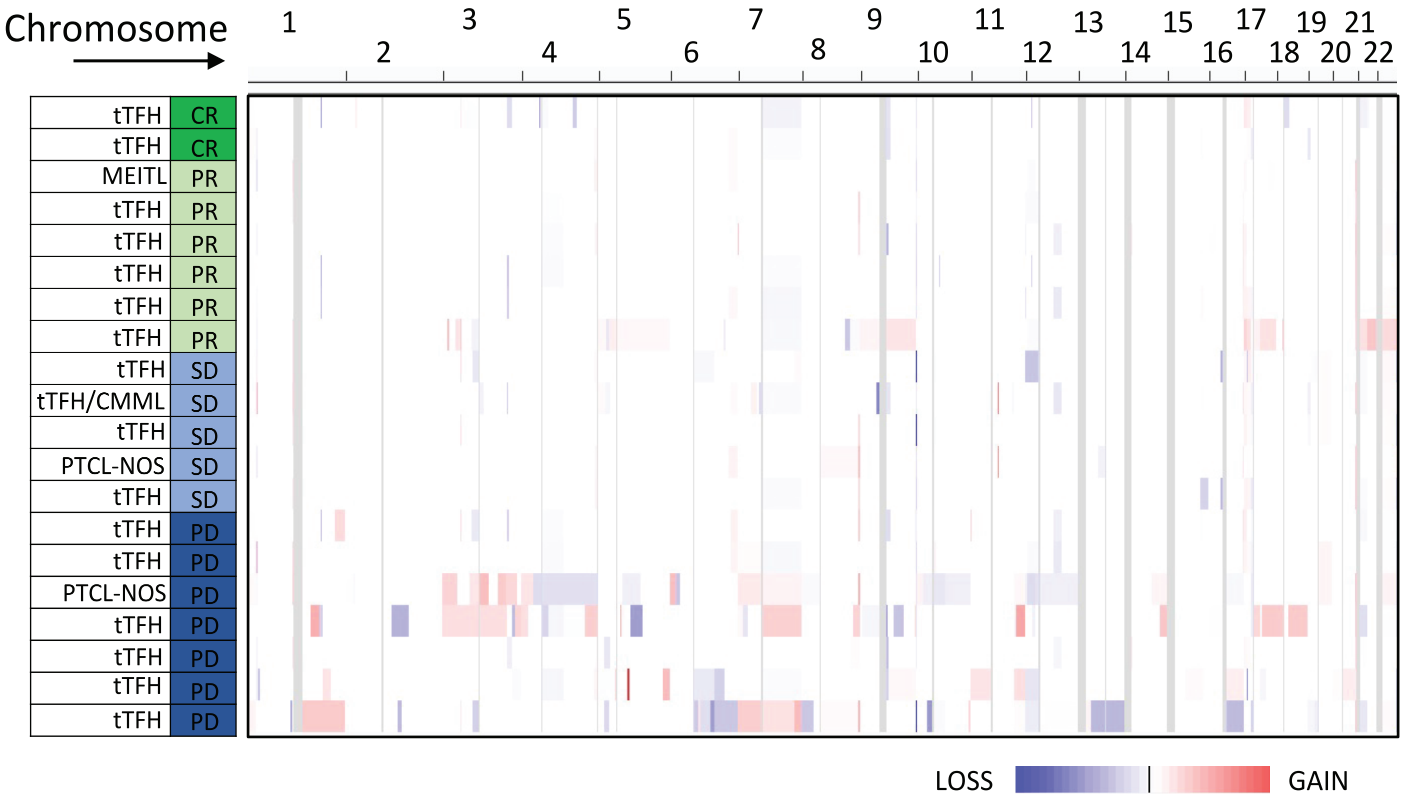

Supplementary Figure S3. Copy number variation (CNV) for pre-treatment plasma DNA samples. Output from CNVkit analysis (log2copy number ratio) is visualized using the Integrative Genomics Viewer with rows representing each patient sample. Genomic loss (blue) and gain (red) are displayed for autosomal chromosomes. Samples are ordered according to best response criteria.

(A)

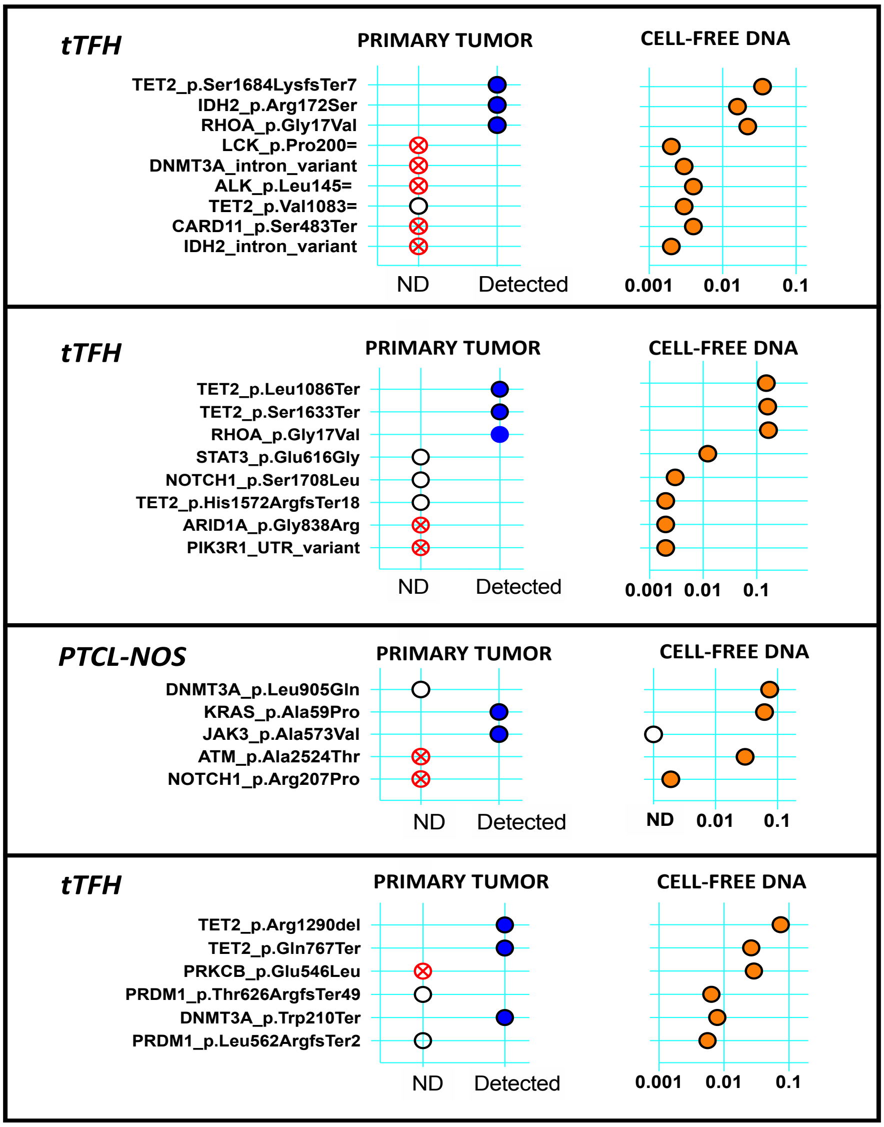

Supplementary Figure S4. Comparison of SNVs detected in cell-free (plasma) at baseline and primary tumor NGS sequencing (4 representative patients). VAFs are represented on right side panels (x-axis) and either detected or not detected (ND, open symbols) for lower depth sequencing of primary tumors (left side panels). Instances for primary tumor sequencing where depth of sequencing was too low to call a variant or genomic areas were not interrogated are represented with red (cross) symbols.

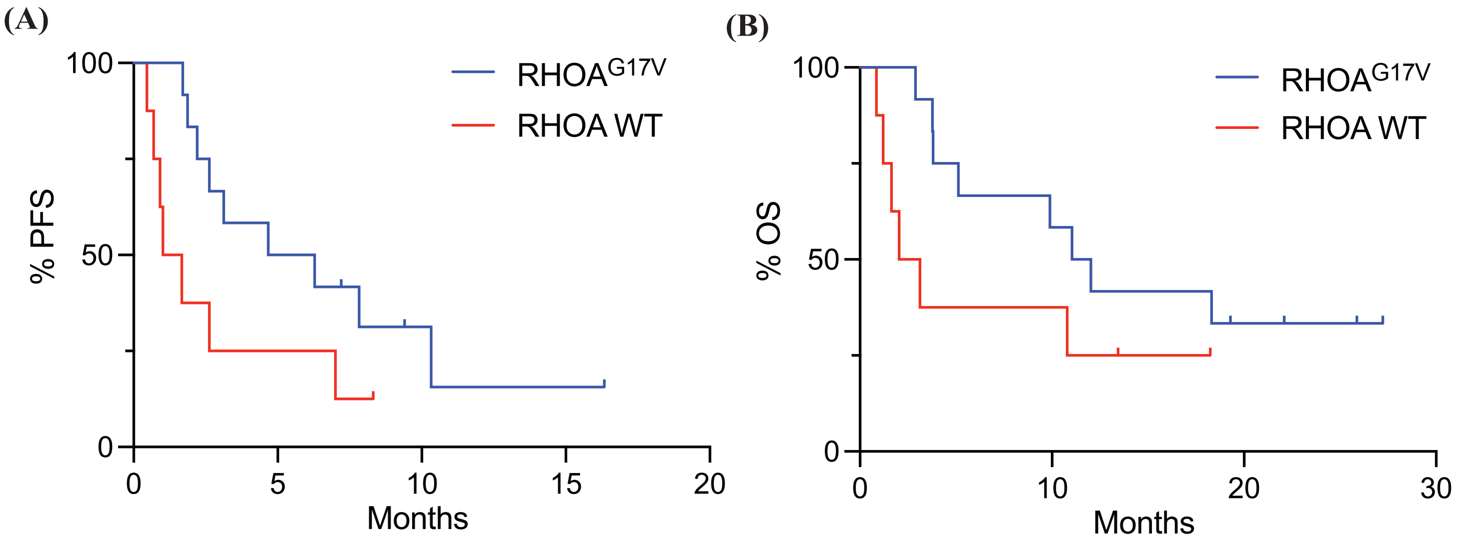

Supplementary Figure S5. (A) Progression free survival (PFS) for guadecitabine-treated patients stratifed by *RHOA* status (mutated versus wild type, WT). Median PFS 5.47 versus 1.35 months; p = 0.02 Wilcoxon, p = 0.06 Log-Rank. **(B)** Overall survival (OS) stratified by *RHOA* status (mutated versus WT). Median OS 11.5 versus 2.58 months; p = 0.05 Wilcoxon, p = 0.15 Log-Rank.

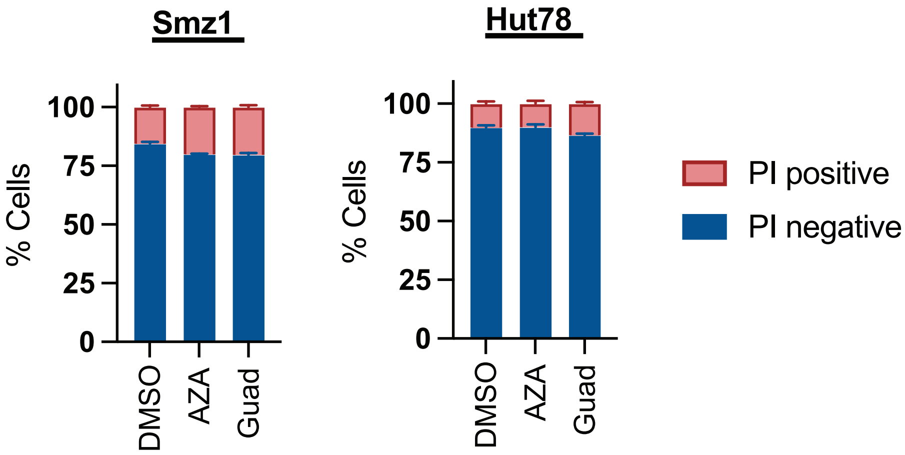

Supplementary Figure S6. Viability (PI exclusion by FACS) of Smz1 and Hut78 cells after culture for 72hrs in vehicle (DMSO), azacitidine (AZA) or guadecitabine (Guad) at 100nM prior to submission for RNA Seq analysis.

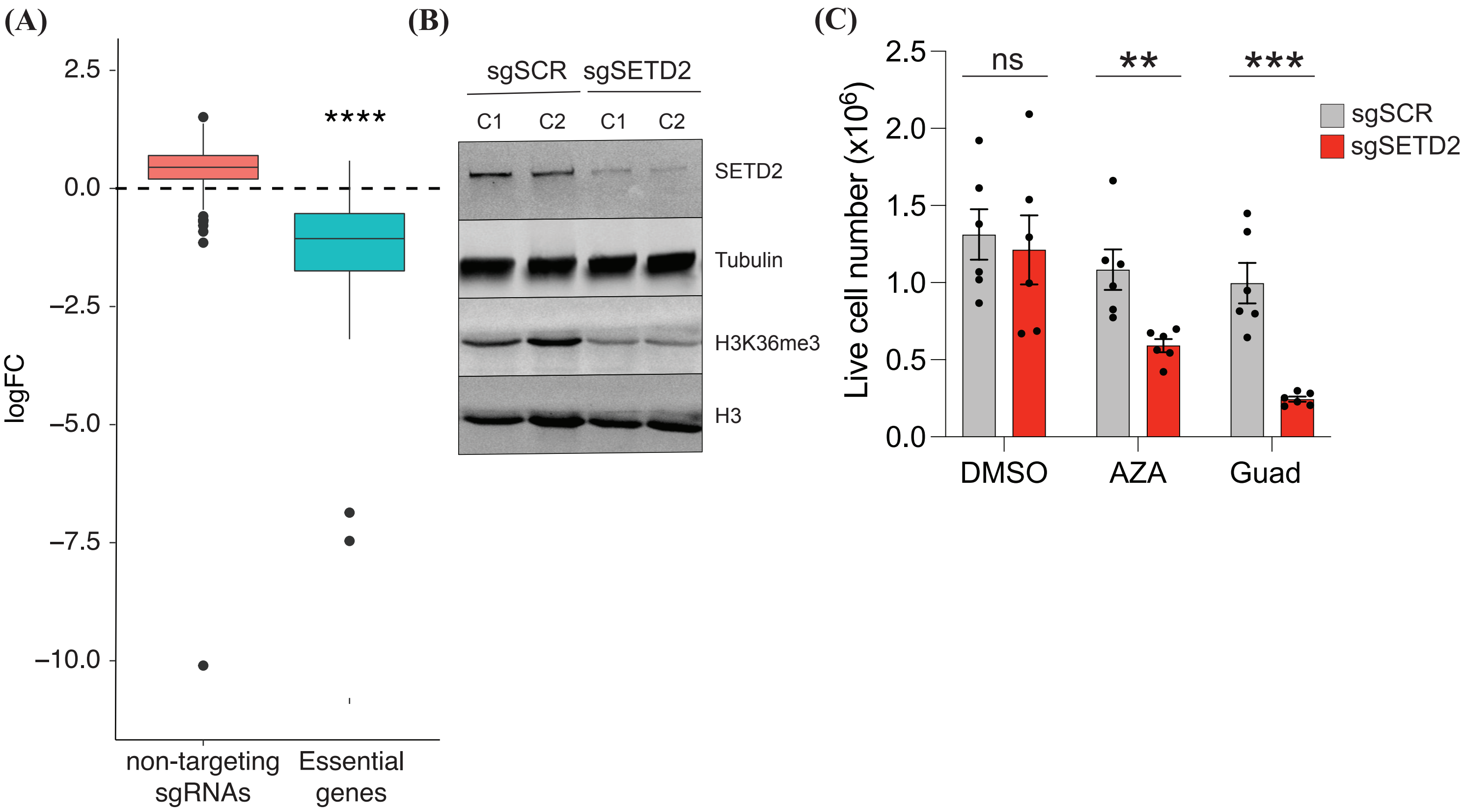

Supplementary Figure S7. (A) sgRNA depleted in DMSO condition (pan-essential cancer genes) **(B)** Western blot of clonal cell lines generated from bulk-transduced Hut78 cells. **(C)** sgSCR_C1 and sgSETD2_C1 cells were treated with DMSO, guadecitabine (100 nM) or AZA (800 nM) for 72 hours and the total viable cells quantified by counting using Trypan blue exclusion. Error bars indicate mean ± s.d. from 6 biological replicates. Significance was analysed using parametric two-tailed student's t-test. ** $p < 0.01$, *** $p < 0.001$.
