# Supplementary material for: Integrated clinical and genomic evaluation of guadecitabine (SGI-110) in peripheral T-cell lymphoma
Source: Leukemia. 2022 Apr 22;36(6):1654–65. doi: 10.1038/s41375-022-01571-8 (PMC9162925; doi:10.1038/s41375-022-01571-8)
Supplement: Supplementary file 2 — Methodology Supplement [file 41375_2022_1571_MOESM2_ESM.docx]

**Methodology Supplement**

*Cell Lines and Reagents*

RPMI-8226, Jurkat, HH and Hut78 were from Prof Ricky Johnstone (Peter MacCallum Cancer Centre, Vic, Australia). Smz1 and MAC2A cells were from Prof David Weinstock (Dana Farber Cancer Institute, MA, USA). OCI-Ly12 and OCI-Ly13.2 cells were from Prof Mark Minden (University Health Network, ON, Canada). All lines were routinely tested for *Mycoplasma*, authenticated by short tandem repeat profiling and cultured at 37^0^C/5% CO_2_ in RPMI1640 medium (Gibco) with 10% fetal bovine serum and pen/strep (Invitrogen). Guadecitabine was provided by Astex Pharmaceuticals Inc. (Pleasanton, CA, USA). AZA (Vidaza, Celgene Pty Ltd, Vic, Australia) was obtained from the Monash Health clinical pharmacy.

*Western blotting*

Lysates were prepared using RIPA buffer with phosphatase and protease inhibitors and separated by SDS-PAGE prior to immunoblotting on PVDF membranes and probing with the following primary antibodies: Abcam: H3K36me3 rabbit pAb (9050); Sigma: α-tubulin mouse mAb (T9026), DNMT1 rabbit mAb (D4692); Cell Signaling: γH2A.X rabbit mAb (9718), SETD2 (E4W8Q) rabbit pAb (80290S), and total H3 (96C10) mouse mAb (3638S).

*Cell death and colony assays*

Cell lines were cultured in drug/vehicle for 3-5 days prior to viability assessment by annexin V staining, propidium iodide (PI) exclusion or cell cycle analysis by nuclear DNA content. For colony assays, cells were treated with drug or vehicle for 72hrs prior to layering on 0.7% agar and incubating 37^0^C. After 21 days, colonies were co-fixed and stained in ethanol/crystal violet prior to counting.

*Lentiviral transduction*

Non-replicating lentiviruses were generated by transient transfection of HEK293T and transduced into Hut78 cells using spinfection at 1,000g for 60 min with 4μg/ml sequabrene (Sigma-Aldrich). pMDL (Addgene #12251), pRSV-REV (Addgene #12253) and pVSVg (Addgene #12259) were used as the packaging plasmids. Cas9-mCherry (Addgene #70182) was used to generate stable Cas9-expressing cells. To generate a custom epigenetics focussed sgRNA library, guide sequences targeting 918 epigenetic regulators (4 sgRNAs/gene) and 328 non-targeting controls (guide sequences are provided in supplementary Table 7) were PCR amplified from a CustomArray Inc oligo pool and cloned into the lentiGuide-Puro vector (Addgene #52963). For proliferative competition assays, SETD2 (CCTGAAGTCATCCATGACAC and TTAAAGAACCAGTTGATACG) and scrambled (GCACTCACATCGGACATCA) sgRNAs were cloned into the lentiGuide-Crimson vector (Addgene #70683).

*Competition Assays*

Hut78/Cas9 cells were transduced with *SETD2* or scrambled sgRNAs encoded on vectors co-expressing a Crimson (Crim) fluorescent reporter and mixed with non-transduced cells. Mixed populations were cultured in the same well with drugs/vehicle refreshed daily for 14 days. The percentage of Crim^+^ were measured every 3–4 days on a FACSCanto II (BD Biosciences) and normalized to day zero.

*RNA sequencing*

Hut78 and Smz1 cells were treated with Guadecitabine (100nM), AZA (100nM) or vehicle (DMSO) for 72 hours in biological triplicate. Cells were lysed in Trizol and RNA extracted using Directzol RNA miniprep kit (Zymo research). Quant-seq 3′mRNA-seq Library Prep Kit for Illumina (Lexogen) was used to generate libraries as per manufacturer’s instructions. Pooled libraries were sequenced with 75 bp single-end sequencing to a depth of 8–11×10^6^ reads on a NextSeq500 (Illumina) at the Peter MacCallum Genomics facility. Sequencing reads were demultiplexed using bcl2fastq (v2.17.1.14) and low-quality reads (Q<30) removed. Reads were trimmed at the 3’ end to remove poly-A-tail derived reads using cutadapt (v2.1), mapped to the reference genome (hg19) using HISAT2 (v2.0.4) and counted using subread (v1.6.3). Read normalization, differential gene expression analysis and figure generation was performed in R(v4) using packages LIMMA(v3.46.0), edgeR(v3.32.1), pheatmap(v1.0.12) and ggplot2(v3.3.3). GSEA(v4.1.0) was used for analysing enrichment of gene sets.

*CRISPR screening*

The screen was performed using recommended protocols described previously^1^ with minor modifications. Briefly, the epigenetics sgRNA library was transduced into Hut78/Cas9 cells at a multiplicity-of-infection of 0.3. 72 hours after addition of the virus, transduced cells were selected for with 1μg/mL puromycin for seven days. A time-point zero (T0), pellets of 8 x10^6^ live cells were harvested, and the remaining cells were split into three treatment conditions: guadecitabine, AZA or DMSO. Drug/vehicle were refreshed daily, and cells passaged every 3–4 days, maintaining 2000x representation by seeding 10 x10^6^ cells in every passage. 8 x10^6^ cells per condition were harvested at endpoint. Genomic DNA was extracted with the DNAase Blood and Tissue kit (Qiagen, Hilden, Germany) and sgRNA sequences PCR amplified in multiple reactions using staggered P5 primers and barcoded P7 primers using Ex Taq DNA Polymerase (Takara; Japan). PCR products were pooled, purified using AMPure XP beads (Beckman Coulter) and sequenced on a NextSeq500 (Illumina) with 75bp single-end sequencing. Reads were demultiplexed using bcl2fastq (v2.17.1.14) and low-quality reads (Q<30) removed. The reads were trimmed using cutadapt (v1.14) to extract the 20bp guide sequence and sgRNAs that were enriched or depleted in response to HMA treatment relative to T0 or DMSO determined using MAGeCK algorithm^2^ (v0.5.6). R packages ggplot2 (v3.3.3) and ggrepel (v0.9.1) were used for figure generation.

*Histological assessment and designation of PTCL subtype*

Cases were classified according to the 2016 revision of the World Health Organisation classification of lymphoid neoplasms (Swerdlow et al, *Blood* 2016; 127: 2375-90). The determination of nodal PTCL with TFH phenotype was adjudicated by central pathology review (BK, JS) on the basis of CD4^+^ tumour cells with at least two TFH related antigens (of BCL6, CD10, CD279/PD1, CXCL13) and/or the presence of a canonical *RHOA^G17V^* mutation (where immunohistochemical markers were equivocal or unavailable).

*Plasma DNA Mutational Analyses*

Input cfDNA for NGS library preparation was 45 to 50 ng, harvested from (on average) 8 mL STRECK plasma (*i.e.,* two STRECK^®^ blood collection tubes). For variant calling, a minimum four Alt reads for the variant in question was required for demultiplexed BAM files, using UMI families with three or more members. Other *VarDict* metrics such as ODDRATIO (values 1 to <1.5) and PMEAN (>19) were used to filter out strand bias and potential end read errors from sequence data^19^. ctDNA burden, expressed as hGE/mL plasma, has been calculated from the measured ng/mL cfDNA concentrations and SNV variant allele frequencies.

**Additional references related to methodology supplement:**

1. Gruber E, Franich RL, Shortt J, Johnstone RW, Kats LM. Distinct and overlapping mechanisms of resistance to azacytidine and guadecitabine in acute myeloid leukemia. *Leukemia*. 2020; **34**: 3388-3392.

2. Li W, Xu H, Xiao T, Cong L, Love MI, Zhang F, et al. MAGeCK enables robust identification of essential genes from genome-scale CRISPR/Cas9 knockout screens. *Genome Biol*. 2014; **15**: 554.
