# Supplementary material for: Integrated clinical and genomic evaluation of guadecitabine (SGI-110) in peripheral T-cell lymphoma
Source: Leukemia. 2022 Apr 22;36(6):1654–65. doi: 10.1038/s41375-022-01571-8 (PMC9162925; doi:10.1038/s41375-022-01571-8)
Supplement: Supplementary file 3 — Supplementary Table S1 [file 41375_2022_1571_MOESM3_ESM.docx]

| *ALK* | *DNMT3A* | *LCK* | *SETD2* |
| --- | --- | --- | --- |
| *ARID1A* | *FYN* | *NCOR1* | *STAT3* |
| *ATM* | *GTF2I* | *NOTCH1* | *STAT5B* |
| *B2M* | *IDH2* | *PIK3R1* | *TET1* |
| *BCOR* | *JAK1* | *PLCG1* | *TET2* |
| *BRAF* | *JAK2* | *PRDM1* | *TET3* |
| *CARD11* | *JAK3* | *PRKCB* | *TP53* |
| *CD28* | *KDM6A* | *PTPN6* | *VAV1* |
| *CD58* | *KRAS* | *RHOA* | *VAV2* |

**Supplementary Table S1.**  Genes represented in ctDNA capture panel. Hybrid capture bait (Agilent Sure Select) design encompassing 36 genes that are recurrently mutated in T-cell lymphoma plus a 1040 single nucleotide polymorphism bait set for copy number variation backbone. Total probe size 435.4 kb.
