# Supplementary material for: Integrated clinical and genomic evaluation of guadecitabine (SGI-110) in peripheral T-cell lymphoma
Source: Leukemia. 2022 Apr 22;36(6):1654–65. doi: 10.1038/s41375-022-01571-8 (PMC9162925; doi:10.1038/s41375-022-01571-8)
Supplement: Supplementary file 4 — Supplementary Table S2 [file 41375_2022_1571_MOESM4_ESM.docx]

| **Gene** | **ORR –**  **mutated** | **ORR –**  **WT/silent** | **ORR**  **P value^†^** | **PFS**  **P value** | | **OS**  **P value** | |
| --- | --- | --- | --- | --- | --- | --- | --- |
|  |  |  |  | **Wilcoxon** | **Log-Rank** | **Wilcoxon** | **Log-Rank** |
| ***TET2*** | 8/16 (50%) | 0/4 (0%) | 0.12 | 0.087 | 0.355 | 0.235 | 0.400 |
| ***DNMT3A*** | 4/12 (33%) | 4/8 (50%) | 0.65 | 0.969 | 0.757 | 0.845 | 0.612 |
| ***IDH2*** | 1/3 (33%) | 7/17 (41%) | 0.99 | 0.800 | 0.957 | 0.999 | 0.962 |
| ***RHOA*** | 7/12 (58%) | 1/8 (13%) | 0.07 | 0.021 | 0.061 | 0.053 | 0.145 |
| ***TP53*** | 4/7 (57%) | 4/13 (36%) | 0.36 | 0.315 | 0.511 | 0.129 | 0.110 |

**Supplementary Table S2.** Response rate by candidate mutation status. WT, wild type. ORR, overall response rate (complete response + partial response); PFS, progression free survival; OS, overall survival; ^†^Fisher’s exact test.
