# Supplementary material for: Integrated clinical and genomic evaluation of guadecitabine (SGI-110) in peripheral T-cell lymphoma
Source: Leukemia. 2022 Apr 22;36(6):1654–65. doi: 10.1038/s41375-022-01571-8 (PMC9162925; doi:10.1038/s41375-022-01571-8)
Supplement: Supplementary file 5 — Supplementary Table S3 [file 41375_2022_1571_MOESM5_ESM.docx]

| **Diagnosis** | **Best response** | **TP53 mutation (hg19) and type** | **COSMIC ID & ClinVar annotation** |
| --- | --- | --- | --- |
| PTCL-TFH | Complete Response | 17:7578457 C>T; missense; R158H 17:7578526; C>T; missense; C135Y | COSM10690; *Pathogenic/Likely pathogenic* COSM10801; *No assignment* |
| AITL | Complete Response | 17: 7578556 T>C; splice site; _126_ | *No entries* |
| AITL | Partial Response | 17: 7577082 C>T; missense; E286K 17:7578262 C>T; missense; R196Q | COSM44599; No assignment  COSM10662; Pathogenic/Likely pathogenic |
| MEITL | Partial Response | 17:7577120 C>A; missense; R273L | COSM10779; *No assignment* |
| AITL + CMML | Stable Disease | 17:7577120 C>A; missense; R273L | COSM10779; *No assignment* |
| PTCL-NOS | Progressive Disease | 17:7577559 G>GA; insertion; S241 (21 amino acid insertion) | *No entries* |
| AITL | Progressive Disease | 17:7577120 C>T; missense; R273H | COSM10660; *Pathogenic/Likely pathogenic* |

**Supplementary Table S3:** Somatic *TP53* mutations detected in pre-treatment cell-free tumor DNA of patient cohort. ID numbers and *TP53* ClinVar designations are listed if assigned.
